# Bacterial Antigen Expression Is an Important Component in Inducing an Immune Response to Orally Administered *Salmonella*-Delivered DNA Vaccines

**DOI:** 10.1371/journal.pone.0006062

**Published:** 2009-06-26

**Authors:** Michelle E. Gahan, Diane E. Webster, Steven L. Wesselingh, Richard A. Strugnell, Ji Yang

**Affiliations:** 1 Department of Microbiology and Immunology, University of Melbourne, Parkville, Victoria, Australia; 2 Children's Vaccines Group, Macfarlane Burnet Institute for Medical Research and Public Health, Melbourne, Victoria, Australia; 3 Department of Medicine, Monash University, Alfred Hospital, Prahran, Victoria, Australia; 4 School of Biological Sciences, Monash University, Victoria, Australia; Queensland Institute of Medical Research, Australia

## Abstract

**Background:**

The use of *Salmonella* to deliver heterologous antigens from DNA vaccines is a well-accepted extension of the success of oral *Salmonella* vaccines in animal models. Attenuated *S. typhimurium* and *S. typhi* strains are safe and efficacious, and their use to deliver DNA vaccines combines the advantages of both vaccine approaches, while complementing the limitations of each technology. An important aspect of the basic biology of the *Salmonella*/DNA vaccine platform is the relative contributions of prokaryotic and eukaryotic expression in production of the vaccine antigen. Gene expression in DNA vaccines is commonly under the control of the eukaryotic cytomegalovirus (CMV) promoter. The aim of this study was to identify and disable putative bacterial promoters within the CMV promoter and evaluate the immunogenicity of the resulting DNA vaccine delivered orally by *S. typhimurium*.

**Methodology/Principal Findings:**

The results reported here clearly demonstrate the presence of bacterial promoters within the CMV promoter. These promoters have homology to the bacterial consensus sequence and functional activity. To disable prokaryotic expression from the CMV promoter a series of genetic manipulations were performed to remove the two major bacterial promoters and add a bacteria transcription terminator downstream of the CMV promoter. *S. typhimurium* was used to immunise BALB/c mice orally with a DNA vaccine encoding the C-fragment of tetanus toxin (TT) under control of the original or the modified CMV promoter. Although both promoters functioned equally well in eukaryotic cells, as indicated by equivalent immune responses following intramuscular delivery, only the original CMV promoter was able to induce an anti-TT specific response following oral delivery by *S. typhimurium*.

**Conclusions:**

These findings suggest that prokaryotic expression of the antigen and co-delivery of this protein by *Salmonella* are at least partially responsible for the successful oral delivery of C-fragment DNA vaccines containing the CMV promoter by *S. typhimurium*.

## Introduction

Oral delivery of DNA vaccines by *Salmonella* was first reported in 1997 [1]. In the decade that followed, *Salmonella* has been used to deliver DNA vaccines targeting a variety of pathogens, and for cancer immunotherapy (for reviews see [2]–[5]). The use of *Salmonella* to deliver heterologous antigens is a natural extension of the success of oral *Salmonella* vaccines. Attenuated *S. typhimurium* and *S. typhi* strains are safe and efficacious [6] and their use to deliver DNA vaccines combines the advantages of both vaccine approaches, while complementing the limitations of each technology.

Previous studies involving DNA delivery by *Salmonella* have commonly used plasmids with gene expression under the control of the cytomegalovirus (CMV) promoter. There have been contrasting reports on the existence of bacterial promoters within the CMV promoter. Studies using green fluorescent protein (GFP) [7], [8] and beta galactosidase (β-gal) [1], [9] have concluded that gene expression occurred in eukaryotic cells but not in *Salmonella,* however, expression was not examined for resultant immunogenicity. In contrast, Goussard *et al.* [10] found expression of lacZ and GFP in *S. typhimurium* and *E. coli* from the CMV promoter. Sequences within the CMV promoter used by bacteria could confound attempts to optimise antigen expression as the resulting immune response might comprise contributions from both eukaryotic and prokaryotic expression, with the extent of each contribution unknown. There have been no published reports examining whether *Salmonella* can be used for the oral delivery of a DNA vaccine in the absence of bacterial expression. The aim of this study was to identify the location of putative bacterial promoters within the CMV promoter, and to construct a DNA vaccine with the bacterial expression component removed, to determine if prokaryotic expression contributes to the immunogenicity of DNA vaccines orally delivered by *S. typhimurium*.

## Methods

### Bacterial strains and plasmids

*S. typhimurium* strain BRD509 is an *aroA*/*aroD* mutant of SL1344 and was the gift of Prof. G. Dougan, Imperial College, London, UK. All DNA manipulations were carried out in *E. coli* (strain JM109 or DH5α). Bacterial strains were routinely cultured in Luria Bertani (LB) or LB agar and when required, were supplemented with 100 µgml^−1^ ampicillin and 25 µgml^−1^ streptomycin. The plasmid pcDNA3/Cfrag, which contains the codon optimised C-fragment of tetanus toxoid under the control of the CMV promoter, was the gift of L. Sait, Department of Microbiology and Immunology, University of Melbourne, Australia. The plasmids pAT153 [11], pTETtac4 containing a C-fragment expression cassette [12], pCR®2.1-TOPO (Invitrogen, Mount Waverley, Australia) and pMu2385 [13] were used in this study.

### Western blot analysis

Whole-cell protein samples were prepared from overnight (O/N) bacterial cultures. Aliquots (300–500 µl) of bacterial culture were centrifuged at 15,000 g for five minutes. The pellet was resuspended in 100 µl of 5× sample loading dye (1 M Tris pH 6.8, 10% SDS, glycerol and 10% (v/v) mercaptoethanol) and boiled prior to loading. Protein samples were separated by 10% SDS-PAGE and transferred to nitrocellulose in blotting buffer (1.44% (w/v) glycine, 0.3% (w/v) Tris and 20% (v/v) methanol) using a Bio-Rad western blotting apparatus at 100 volts for one hour. The nitrocellulose was blocked for one hour at room temperature with 5% skim milk in phosphate buffered saline (PBS), incubated with primary antibody diluted 1/200–1/1000 in PAT (PBS containing 0.05% Tween20 and 0.5% skim milk powder) O/N at room temperature. Following incubation with an anti-mouse Ig affinity isolated horse radish peroxidase conjugated secondary antibody (Chemicon, Temecula, CA, USA) diluted 1/1000 in PAT at room temperature for two hours, proteins were visualised by TMB Membrane Peroxidase Substrate (KPL, MD, USA). The primary antibody was an anti-tetanus toxin antibody generated by subcutaneously immunising five BALB/c mice with 100 µl Tetanus Toxin (CSL Limited, Parkville, VIC, Australia) on day 0 and 49. The sera was collected and pooled on day 56.

### RNA extraction

Total cellular RNA was extracted from early-log phase bacterial cultures using the Qiagen RNeasy mini kit (QIAGEN, Doncaster, VIC, Australia). RNA concentration and purity was determined by spectrophometry.

### Primer labelling with ^32^P

An oligonucleotide (5′-AACTCCCATTGACGTCAATGGG-3′) was radiolabelled at the 5′ end in a 20 µl reaction containing 70 mM Tris pH 7.6, 10 mM MgCl_2_, 5 mM dithiothreitol (DTT), 1 µl[γ-^32^P]ATP and one unit of T4 polynucleotide kinase. Incubation was carried out at 37°C for 30 minutes, followed by heat inactivation at 70°C for 15 minutes.

### Primer extension

Hybridisation of a [γ-^32^P]ATP-labelled oligonucleotide to RNA, followed by extension of the primer using AMV reverse transcriptase (Promega, Sydney, NSW, Australia), was carried out according to the method of Hudson and Davidson [14]. Briefly, the oligonucleotide (50,000–100,000 CPM) was hybridised to 150 µg/ml of RNA in the presence of 56 mM Tris pH 7.5, 20 mM MgCl_2_ and 31.8 mM DTT in a total volume of 50 µl, by heating to 75°C for two minutes and slow cooling to room temperature. Following ethanol precipitation, the pellet was resuspended in 10 µl of 50 mM Tris pH 8.8, 10 mM MgCl_2_, 140 mM KCl, 10 mM DTT, 4 mM tetrasodium pyrophosphate and 100 µM each of the four dNTPs. The primer was extended with five units of reverse transcriptase for two hours at 42°C. After ethanol precipitation, the samples were resuspended in formamide dye mix, denatured and loaded onto a 6% sequencing gel.

### β-galactosidase assay

Plasmid pMu2385 is a single copy plasmid which carriers a promoter-less *lacZ* structural gene with its own translational start signals [13]. CMV promoter regions were excised from the corresponding pcDNA3/Cfrag constructs with *Bgl* II and *Hin*d III and ligated into pMu2385 digested with *Bam*H I and *Hin*d III. Cultures of *E. coli* (DH5α) containing the β-galactosidase vector were grown in LB at 37°C shaking to mid-exponential phase (OD_600_ of 0.4–0.6) and β-galactosidase activity was assayed as described by Miller [15]. Assays were performed in duplicate on at least three separate occasions.

### Ethics statement

All animals were handled in strict accordance with good animal practice as defined by the relevant national and/or local animal welfare bodies, and all animal work was approved by the University of Melbourne animal ethics committee.

### Intramuscular immunisation

Female 6–8 week old BALB/c mice were obtained from The University of Melbourne, Department of Microbiology and Immunology animal facility (Parkville, Victoria, Australia). Endotoxin free DNA vaccines, obtained using EndoFree® Plasmid kits (QIAGEN, Doncaster, VIC, Australia), were diluted to 1 µg/µl in 0.9% sterile saline. 50 µg of DNA was injected intramuscularly in both hind legs of the mice (100 µg total dose).

### Oral immunisation with *S. typhimurium*

Mice were orally immunised under anaesthesia (Penthrane) via a 4 cm gastric lavage needle with approximately 10^10^ bacteria in PBS (200 µl/mouse) as determined by retrospective viable count. Bacteria were grown static in LB containing antibiotics at 37°C for 24 h. Thirty minutes prior to immunisation mice were orally administered 100 µl of 10% (w/v) sodium bicarbonate to neutralise stomach acidity.

### Measurement of antibody response by enzyme-linked immunosorbent assay (ELISA)

On designated days mice were bled from the tail vein and the titre of antibody present for *S. typhimurium* LPS and TT was determined by end-point ELISA as previously described [16]. Total Ig was detected by the addition of 1/1000 anti-mouse Ig Horseradish peroxidase conjugate (Chemicon, Temecula, CA, USA). Serum antibody titres were designated as the reciprocal of the dilution of antibody that gave an OD_492_ value five times the background of the assay.

### Statistical analysis

The non-parametric Mann Whitney test was used to compare groups of data. A probability (P) value of less than 0.05 indicated statistical significance. To perform statistical analysis on data groups which contained values below the point of detection of the assay, an arbitrary value one below the limit of the assay was assigned.

## Results

### Expression of C-fragment in *S. typhimurium* BRD509

**Figure 1:**
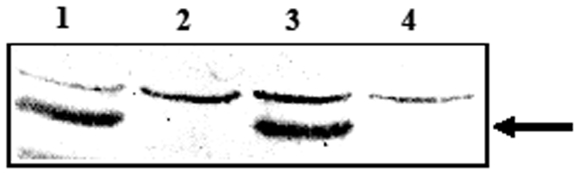
Expression of C-fragment in attenuated *S. typhimurium* BRD509. Western immunoblots showing expression of C-fragment encoded on various plasmids. Cell lysates of (1) BRD509 (pTETtac4), (2) BRD509, (3) BRD509 (pcDNA3/Cfrag), and (4) BRD509 (pcDNA3) probed with polyclonal anti-tetanus toxin antiserum. C-fragment expression is indicated by the arrow.

To determine if C-fragment protein was produced from the DNA vaccine plasmids, total protein from overnight cultures of *S. typhimurium* BRD509 was analysed by western blot. Two protein species were detected (figure 1). A non-specific band at ∼55 kDa was present in all samples, and the C-fragment protein at ∼47.5 kDa was detected for BRD509 containing pTETtac4 and pcDNA3/Cfrag but not for pcDNA3 or BRD509 alone. These results indicate that bacterial promoter activity, either within, or upstream of, the CMV promoter caused read-through transcription into the C-fragment gene resulting in bacterial expression.

### Identification and location of bacterial promoter sequences within the CMV promoter

**Figure 2:**
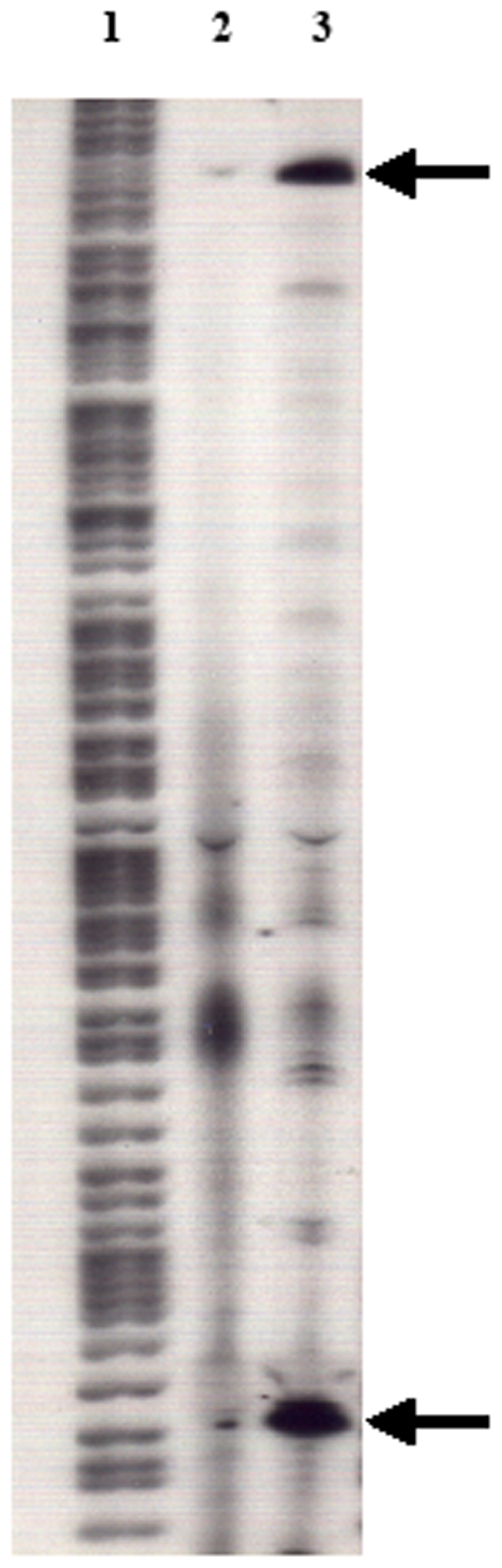
Determination of *S. typhimurium* transcription initiation sites within the CMV promoter by primer extension. A 22-base oligonucleotide primer labelled with P^32^ was hybridised to RNA from BRD509 (lane 2) and BRD509 (pcDNA3/Cfrag) (lane 3) and extended with reverse transcriptase. The products, along with a sequencing ladder generated from pcDNA3/Cfrag (lane 1), were run on a denaturing gel. The primary bacterial transcription initiation sites are indicated with arrows.

**Figure 3:**
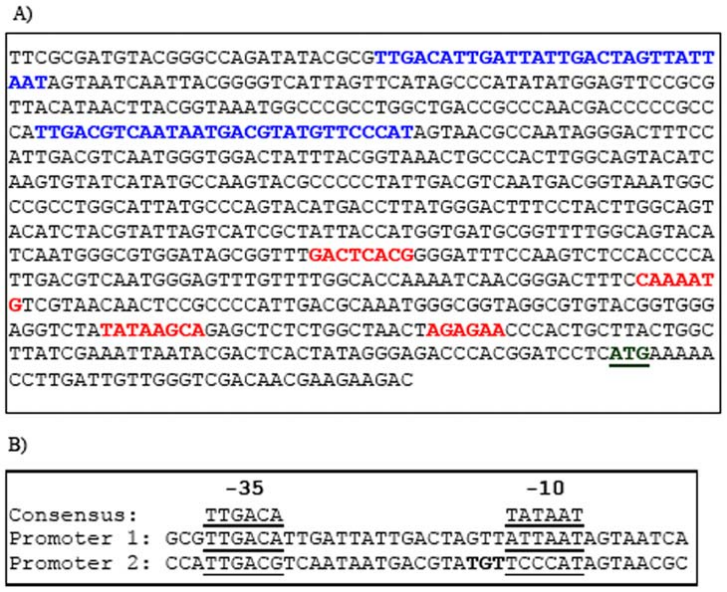
Location of *S. typhimurium* promoter sequences within the CMV promoter. (A) DNA sequence of pcDNA3/Cfrag from the start of the CMV promoter highlighting the location of the two primary bacterial promoters (blue), the regulatory regions of the CMV promoter (red) and the start codon of the C-fragment gene (underlined). (B) Alignment of the −35 and −10 promoter regions of the two primary *S. typhimurium* promoters with the bacterial promoter consensus sequence. The TGT enhancer region located within promoter 2 is shown in bold.

The transcription initiation sites of the bacterial promoter sequences were identified by primer extension analysis using RNA extracted from BRD509 (pcDNA3/Cfrag) and BRD509. Two strong primary bacterial transcription initiation sites situated within the CMV promoter and multiple minor promoters were identified (figure 2). The first of these (promoter one, P1) is located 428 base pairs (bp) upstream of the AP1 region of the CMV promoter and the second promoter (promoter two, P2) is 292 bp upstream of AP1. Both bacterial promoters are located upstream of the CMV promoter regulatory elements and the C-fragment start codon (figure 3a). Comparison of the −35 and −10 regions of the promoters with the bacterial consensus sequence revealed a perfect alignment for the P1 promoter at the −35 position and only two nucleotide changes in the −10 sequence (figure 3b). In addition, the P1 sequence displayed optimal spacing of 17 bp between the −35 and −10 sequence elements. Promoter two contained a near perfect −35 region and an optimal spacer of 17 bp. Although the −10 region is less conserved, there is a TGT motif one base upstream of the −10 region which is known to enhance transcription [17], [18].

### Determination of bacterial promoter activity following removal of the two primary bacterial promoters

**Figure 4:**
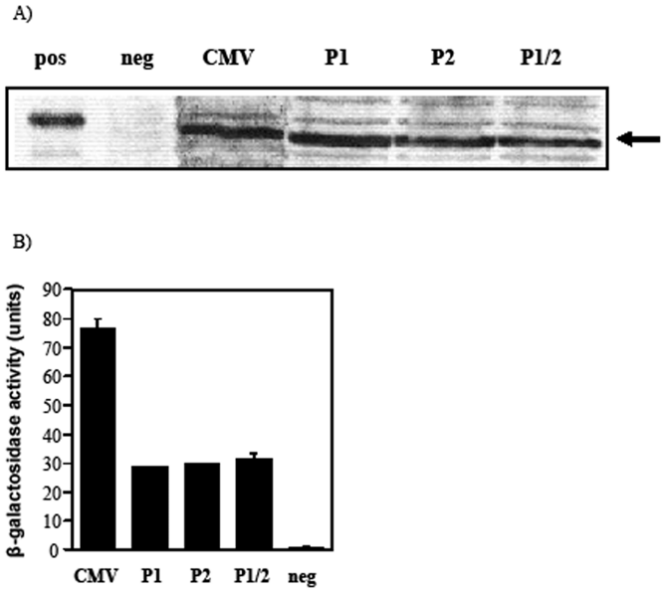
Bacterial promoter activity of the original and mutated CMV promoter. (A) Western immunoblots showing expression of C-fragment from *S. typhimurium* BRD509 containing pcDNA3/Cfrag (CMV), pcDNA3/Cfrag-P1 (P1), pcDNA3/Cfrag-P2 (P2), or pcDNA3/Cfrag-P1/2 (P1/2). Purified C-fragment (pos) and BRD509 alone (neg) were included as controls. (B) To quantify promoter activity, the unmodified pCMV (CMV), pCMV-P1 (P1), pCMV-P2 (P2) and pCMV-P1/2 (P1/2) were cloned into the β-galactosidase vector and promoter activity expressed as units of β-galactosidase activity. Activity of the empty vector (neg) is included as a negative control.

C-fragment plasmids were constructed using PCR and restriction enzyme digests with either P1 (pcDNA3/Cfrag-P1), P2 (pcDNA3/Cfrag-P2) or both bacterial promoter regions removed (pcDNA3/Cfrag-P1/2). To determine if bacterial C-fragment expression had been eliminated, total protein from overnight cultures of *S. typhimurium* BRD509 was analysed by western blot. C-fragment protein expression was detected for BRD509 containing pcDNA3/Cfrag, pcDNA3/Cfrag-P1, pcDNA3/Cfrag-P2 and pcDNA3/Cfrag-P1/2 but not for BRD509 alone (figure 4a). The degree of protein expression for the three modified constructs appeared to be similar to pcDNA3/Cfrag which contains the unmodified CMV promoter.

To determine if differences between the constructs were masked by the high copy number of pcDNA3, CMV-*lacZ* transcriptional fusions of the pCMV, pCMV-P1, pCMV-P2 and pCMV-P1/2 promoter regions were constructed to quantify bacterial promoter activity. The single promoter mutations (pCMV-P1 and pCMV-P2) resulted in an approximately 2.6 fold reduction in β-galactosidase activity compared with the unmodified CMV promoter, which produced 76 units of β-galactosidase activity (figure 4b). There was no further reduction in promoter activity when both primary bacterial promoters were mutated (pCMV-P1/2). Although removal of the two primary bacterial promoter sequences resulted in a reduction in promoter activity, it did not eliminate bacterial expression. For the analysis of *S. typhimurium* cultures this could be due to the additional minor bacterial promoters identified within the CMV promoter (figure 2). Additionally, studies have also identified different bacterial promoters within the CMV promoter which are utilised by *E. coli* [25] and these are likely to account for the results of the β-galactosidase quantification assay which was done in *E. coli*.

### Determination of bacterial promoter activity following insertion of a bacterial transcription terminator

**Figure 5:**
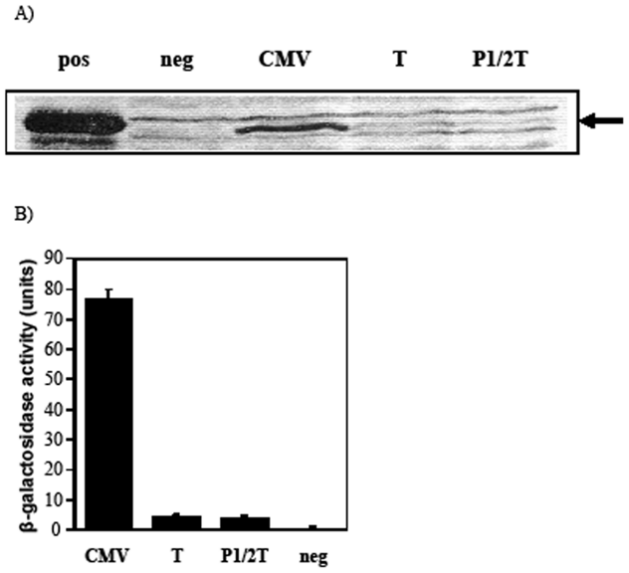
Bacterial promoter activity following insertion of the *trpA* bacterial transcription terminator. (A) Western immunoblots showing expression of C-fragment from *S. typhimurium* BRD509 containing pcDNA3/Cfrag (CMV), pcDNA3/Cfrag-T (T) or pcDNA3/Cfrag-P1/2-T (P1/2T). Purified C-fragment (pos) and BRD509 alone (neg) were included as controls. (B) To quantify promoter activity, the unmodified pCMV (CMV), pCMV-T (T) and pCMV-P1/2-T (P1/2T) were cloned into the β-galactosidase vector and promoter activity expressed as units of β-galactosidase activity. Activity of the empty vector (neg) is included as a negative control.

As it was impractical to remove or mutate all the minor bacterial promoters, the *trpA* bacterial transcription terminator [19] was inserted into pcDNA3/Cfrag and pcDNA3/Cfrag-P1/2 at the junction between the 3′ end of the CMV promoter and the 5′ start of the C-fragment gene. These constructs were designated pcDNA3/Cfrag-T and pcDNA3/Cfrag-P1/2-T. To determine if bacterial protein expression had been eliminated following insertion of the *trpA* terminator total protein from overnight cultures of *S. typhimurium* BRD509 was analysed by western blot. Strong C-fragment protein expression was detected from pcDNA3/Cfrag. In contrast, bacterial expression from pcDNA3/Cfrag-T and pcDNA3/Cfrag-P1/2-T was negligible (figure 5a).

To quantify bacterial promoter activity the pCMV, pCMV-P1/2-T and pCMV-T regions were ligated into pMu2385. The inclusion of the bacterial terminator resulted in a 17-fold reduction in β-galactosidase activity from 76 units for the unmodified CMV promoter to 4.5 units for both pCMV-P1/2-T and pCMV-T. Although substantially reduced, the β-galactosidase activity was still greater than the 0.625 units of β-galactosidase activity detected for the empty vector (figure 5b).

### Humoral immune response following intramuscular immunisation of the modified CMV promoter constructs

**Figure 6:**
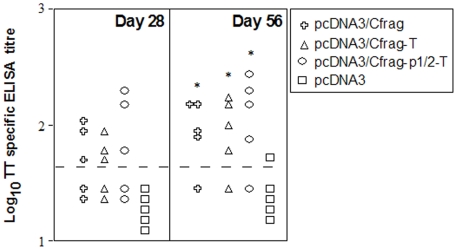
Tetanus toxoid specific serum antibody response following intramuscular DNA immunisation. Groups of five BALB/c mice were intramuscularly immunised with pcDNA3/Cfrag, pcDNA3/Cfrag-T, pcDNA3/Cfrag-P1/2-T or pcDNA3 on days 0 and 7. The tetanus toxin (TT) specific serum total Ig antibody titres were determined by endpoint ELISA. Each symbol represents a titre from an individual mouse. The broken line indicates the limit of the assay (1/50). * = significantly different from the pcDNA3 group (P<0.05).

Groups of five BALB/c mice were intramuscularly injected with pcDNA3, pcDNA3/Cfrag, pcDNA3/Cfrag-T and pcDNA3/Cfrag-P1/2-T DNA on days 0 and 7 to determine *in vivo* functionality and ability to induce an anti-TT antibody response. Four of the five mice immunised with pcDNA3/Cfrag, pcDNA3/Cfrag-T and pcDNA3/Cfrag-P1/2-T responded to the DNA vaccines at day 56 with average titers of 118, 121 and 175 respectively (figure 6). One mouse immunised with pcDNA3 also responded with a titer of 65. There was no significant difference between titres of mice immunised with pcDNA3/Cfrag, pcDNA3/Cfrag-T and pcDNA3-P1/2-T and all groups were significantly higher than the negative control group which received pcDNA3 (P<0.05).

### Humoral immune response following oral delivery of modified CMV promoter constructs by *S. typhimurium* BRD509

Prior research has shown that pAT153 is stable *in vitro* and *in vivo* and is the optimal plasmid for oral delivery by *S. typhimurium* [16]. Therefore, the C-fragment vaccine cassette from pcDNA3/Cfrag-P1/2-T and pcDNA3/Cfrag-T was cloned into pAT153 to generate pAT153/Cfrag-P1/2T and pAT153/Cfrag-T. Groups of five BALB/c mice were orally immunised with *S. typhimurium* BRD509 containing the plasmids pAT153, pAT153/Cfrag, pAT153/Cfrag-T and pAT153/Cfrag-P1/2-T. Mice were immunised on days 0 and 35 and their serum antibody response (total Ig) specific for *S. typhimurium* LPS and tetanus toxoid (TT) was determined by ELISA at days 28 and 56.

**Figure 7:**
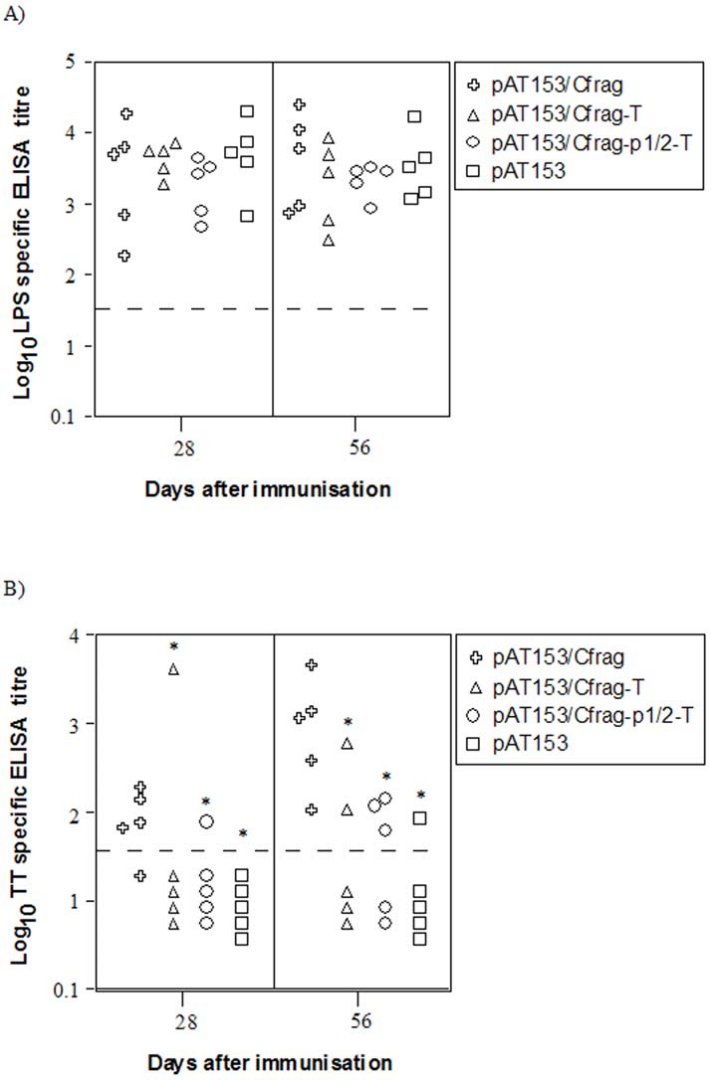
*S. typhimurium* LPS and tetanus toxoid specific serum Ig antibody responses. Groups of five BALB/c mice were orally immunised on day 0 and 35 with attenuated *S. typhimurium* BRD509 containing the plasmids pAT153/Cfrag, pAT153/Cfrag-T, pAT153/Cfrag-P1/2-T or pAT153. Serum samples were collected on days 28 and 56. The amount of (A) *S. typhimurium* LPS-specific and (B) tetanus toxoid (TT)-specific total serum Ig was determined by endpoint ELISA. Each symbol represents a titre from an individual mouse. The broken line indicates the limit of the assay (1/50). * = significantly different from pAT153/Cfrag (P<0.05).

Anti-LPS total Ig was detected in the sera of all immunised mice (figure 7a). The pattern and titre of the antibody response was similar in all groups of mice. There was no significant difference in LPS responses between the groups, nor was there a difference between days 28 and 56, indicating all groups received the same dose of *Salmonella*. At day 56, the anti-TT antibody titres for pAT153/Cfrag were significantly higher than both pAT153/Cfrag-T and pAT153/Cfrag-P1/2-T with average titres of 1652, 181 and 93, respectively. There was no difference between pAT153/Cfrag-T and pAT153/Cfrag-P1/2-T and the negative control group who received pAT153 alone (figure 7b).

## Discussion

DNA vaccines have undergone many revisions and improvements to reduce the required dosing, increase potency, and ultimately reach commercialisation [20]. At the heart of this process has been an ongoing research effort to better understand the basic biology of DNA vaccines. The oral delivery of DNA vaccines by *Salmonella* requires further optimisation if it is to be commercialised. An important aspect of the basic biology of the *Salmonella*/DNA vaccine platform is the relative contributions of prokaryotic and eukaryotic expression in production of the vaccine antigen. We examined whether an immune response can be induced to a DNA vaccine delivered by *Salmonella* in the absence of bacterial antigen expression. Therefore, the aim of this study was to identify and disable putative bacterial promoters within the CMV promoter and evaluate the immunogenicity of the resulting DNA vaccine delivered orally by *S. typhimurium*.

Substantial amounts of C-fragment protein were detected in cultures of *S. typhimurium* containing pcDNA3/Cfrag, a DNA vaccine construct under the control of the CMV promoter. This confirms the presence of bacterial promoters within the CMV promoter sequence. Furthermore, quantification of bacterial expression from the CMV promoter in an *E. coli* β-galactosidase assay indicates that the amount of recombinant protein produced by *E. coli* from the CMV promoter is similar to that reported for other bacterial promoters [21], [22]. Our results are consistent with the study by Goussard *et al.* [10] who detected expression of GFP and lacZ from the CMV promoter in *S. typhimurium* SL7207 and *E. coli.* Interestingly, they found reporter gene expression to be higher in the stationary phase of bacterial growth than the exponential growth phase [10].

Primer extension analysis identified two primary and numerous minor transcription initiation sites within the CMV promoter which are utilised by *S. typhimurium*. The strong activity of the primary bacterial promoters, P1 and P2, are most likely a consequence of their homology to the bacterial promoter consensus sequence [23]. Of the 12 positions in the consensus sequence, most mRNA promoters match from seven to nine bases [24]. The P1 and P2 promoters contain 10 and eight matching bases respectively. Studies by Lewin *et al*. [25] using the rapid amplification of 5′ complementary DNA ends (5′RACE) method and plasmids containing the *lux* gene from *Vibrio harveyi*, have identified two bacterial promoters within the CMV promoter utilised by *E. coli*. These promoters showed homology to the bacterial −10 and −35 consensus sequences and resulted in strong expression of a bacterial cytotoxin. Interestingly, the promoters identified by Lewin *et al*. in *E. coli* were situated in the later part of the CMV promoter, in contrast to the location of the promoters utilised by *S. typhimurium* which were mapped to early in the CMV promoter region. This suggests there are multiple regions in the CMV promoter from which bacterial expression can occur and that different bacterial species may preferentially utilise specific promoters.

There are very few reports addressing the relative contribution of prokaryotic and eukaryotic expression to the target-specific immune response. DNA vaccines with prokaryotic promoters are ineffective when delivered by *S. typhimurium*, which indirectly suggests that immune responses can be attributed to the use of eukaryotic promoters [1], [7], [9]. Additionally, *S. typhimurium* studies comparing the expression of GFP constructs with and without introns, also support transcription of the target protein by eukaryotic cells [8]. While these reporter gene studies support the efficacy of eukaryotic transcription, they do not preclude a contribution from prokaryotic expression when the CMV promoter is used with *Salmonella*. In contrast, other studies have shown expression of C-fragment protein from the CMV promoter in lysates of *S. typhi* vaccine strain CVD915 [26] and attenuated *Shigella flexneri* 2a vaccine strain CVD1204 [27] under both aerobic and non-aerobic conditions. Under aerobic conditions in lysates of *S. typhi* CVD915 the level of prokaryotic expression of C-fragment from the DNA vector was similar to that of the corresponding protein vector as determined by western blot [26]. Despite this, both reports argued that the immune response from the DNA plasmid was primarily a consequence of transcription by the eukaryotic host rather than the bacterial vector [26], [27].

To directly evaluate the impact of prokaryotic expression on the immune response induced by DNA vaccines delivered by *Salmonella*, a series of genetic manipulations were performed to eliminate promoter expression in *S. typhimurium*, while maintaining activity of the CMV promoter in mammalian cells. Removal of the primary bacterial promoters, P1 and P2, was insufficient to eliminate prokaryotic transcription, presumably due to the presence of additional bacterial promoters. Although small deletions within the CMV promoter do not affect promoter activity, large deletions may [28]. Therefore, the *trpA* bacterial transcription terminator was inserted at the junction between the CMV promoter and the C-fragment gene, thereby abrogating the activity of any bacterial promoters situated within the CMV promoter. The inclusion of the terminator resulted in a significant reduction in protein expression from pcDNA3/Cfrag-P1/2-T in the lysates of *S. typhimurium* BRD509. Furthermore, there was no significant difference in anti-TT antibody titres following intramuscular injection of pcDNA3/Cfrag-P1/2-T and pcDNA3/Cfrag-T compared with pcDNA3/Cfrag, indicating that these DNA vaccines retain comparable function in eukaryotic cells and are capable of eliciting similar anti-TT antibody responses.

The final step was to evaluate the modified CMV promoter in the context of an optimised *Salmonella*/DNA oral delivery platform. The vaccine cassettes were transferred into pAT153 which has previously been shown to be the optimal plasmid vector for oral delivery by *S. typhimurium* [16]. Following oral delivery by *S. typhimurium* BRD509, the only construct capable of inducing a significant anti-TT antibody response was pAT153/Cfrag, which contains the unmodified CMV promoter. Compared with the negative control no significant anti-TT responses were recorded for mice immunised with pAT153/Cfrag-P1/2-T or pAT153/Cfrag-T. These findings suggest that successful oral delivery of C-fragment DNA vaccines by *S. typhimurium* may be partially dependant on prokaryotic expression of the antigen and co-delivery of this protein by *Salmonella*.

DNA vaccines, processed through the MHC I pathway, generate a cell-mediated biased immune response. In contrast, protein vaccines are processed through the MHC II pathway and produce an antibody biased immune response. As *Salmonella* delivered DNA vaccines have a DNA and protein component, it is plausible that both MHC I and II processing will be employed which may enhance vaccine immunogenicity. Further work is required to elucidate and understand the mechanisms of prokaryotic and eukaryotic antigen processing and presentation following DNA delivery by *Salmonella*. By altering the ratio of MHC I and II processing, vaccine strategies could be designed to modulate the immune response generated by a vaccine, thereby more effectively targeting a specific pathogen.

The data presented in this study suggest bacterial antigen expression may be an important component of inducing an immune response to a DNA vaccine orally delivered by *S. typhimurium*. Additional studies are required to determine the extent to which these findings can be extrapolated to other bacterial species, target antigens, doses and routes of immunisation. This will enable accurate characterisation of the vaccine for commercial licensing and further optimisation by modification of the ratio of eukaryotic and prokaryotic expression to improve vaccine immunogenicity. Additionally, bacterial gene expression from DNA vaccine constructs raises important safety implications when handling the modified bacteria, particularly if it contains virulence or toxin genes. The findings of our study have important implications for the development, optimisation and commercialisation of the *Salmonella*/DNA vaccine platform.
